# Early Risk Assessment and Recognition of Allergies in Children: Rationale, Methodology, and Proposed Algorithms

**DOI:** 10.1111/all.70224

**Published:** 2026-01-25

**Authors:** E. Hamelmann, B. Schaub, K. Beyer, K. Blümchen, M. Gerstlauer, M. V. Kopp, L. Lange, S. Lau, N. Maison, H. Ott, S. M. Schmidt, T. Spindler, C. Traidl‐Hoffmann, C. Vogelberg

**Affiliations:** ^1^ Department of Pediatrics, Children's Center Bethel, University Hospital OWL University Bielefeld Bielefeld Germany; ^2^ Pediatric Allergology, Department of Pediatrics, Dr. Von Hauner Children's Hospital University Hospital Munich Germany; ^3^ Member of German Center for Lung Research—DZL, LMU Munich Germany; ^4^ Member of German Center for Child and Adolescent Health—DZKJ, LMU Munich Germany; ^5^ Ludwig‐Maximilians‐Universität Munich Munich Germany; ^6^ Department of Pediatric Respiratory Medicine, Immunology and Critical Care Medicine Charité—Universitätsmedizin Berlin Berlin Germany; ^7^ Member of German Center for Child and Adolescent Health (DZKJ)‐Partner Site Berlin Berlin Germany; ^8^ Department of Pediatrics, Division of Pneumology, Allergology, Gastroenterology and Infectious Diseases Goethe University Frankfurt Germany; ^9^ Paediatric and Adolescent Medicine, Department of Paediatric Pneumology and Allergology University Medical Center Augsburg Augsburg Germany; ^10^ Department of Paediatrics, Inselspital, Bern University Hospital University of Bern Bern Switzerland; ^11^ GFO Kliniken Bonn Bonn Germany; ^12^ Institute of Asthma‐ and Allergy Prevention (IAP), Helmholtz Zentrum Munich German Research Center for Environmental Health (GmbH) Neuherberg Germany; ^13^ Section of Pediatric Dermatology, Munich University Center for Children With Medical and Developmental Complexity Ludwig‐Maximilians‐University Hospital Munich Germany; ^14^ Department of Pediatrics University Medicine Greifswald Germany; ^15^ Allergologisch‐Pneumologische Ambulanz Pädiatrie Medizincampus Bodensee Friedrichshafen Germany; ^16^ Departement of Pediatrics—Pneumology, Allergology Klinikum Kempten Kempten Germany; ^17^ Medical Faculty, Institute of Environmental Medicine and Integrative Health, EMIH University Augsburg Augsburg Germany; ^18^ Department of Pediatrics, Faculty of Medicine and University Hospital Carl Gustav Carus Technische Universität Dresden Dresden Germany

**Keywords:** atopic diseases, children, early allergy recognition, predictive markers, prevention, risk assessment, risk factors

## Abstract

**Background:**

Atopic diseases—including atopic dermatitis (AD), food allergy (FA), allergic rhinitis (AR), and asthma—are the most common chronic conditions in childhood and adolescence, affecting up to 30% of the global population. In Germany alone, more than 2.1 million children and adolescents are affected. These conditions frequently coexist and share common genetic, environmental, dietary, and microbial risk factors.

**Methods:**

A comprehensive literature review was conducted, and a multidisciplinary Task Force of the German Society for Pediatric Allergology and Environmental Medicine (GPA) and the German Society for Allergology and Clinical Immunology (DGAKI) developed consensus‐based algorithms for early risk assessment and recognition of allergies in children in already existing preventive medical check‐ups. The approach emphasizes stepwise risk assessment, including family history, environmental exposures, and early clinical signs such as recurrent wheezing.

**Results:**

For children identified as at risk for or with early clinical signs of atopy, targeted diagnostic steps are recommended that follow the national/international guidelines for the management of the suspected atopic disease. This may include general and specific recommendations for nutrition, measurement of specific sensitization and selected biomarkers, if indicated and recommended by the guidelines. Routine allergy testing in asymptomatic children is not recommended. The algorithms are designed to be embedded into routine pediatric check‐ups, enabling systematic and early identification of children at increased risk or with early clinical signs of atopic diseases. Early recognition and management can reduce disease severity, improve quality of life, and decrease healthcare costs.

**Conclusion:**

Structured programs for early risk assessment and recognition of allergies in children are currently lacking but can provide substantial clinical and economic benefits. Integration into routine pediatric preventive examinations, supported by standardization, interdisciplinary collaboration, and sustainable funding, offers a promising strategy to improve long‐term outcomes for affected children and their families.

## 1 Background and Objective

### 1.1 Allergies and Their Relevance for Public Health

Allergic and atopic diseases—including atopic dermatitis (AD), IgE‐mediated food allergy (FA), allergic rhinitis (AR), and allergic asthma (AA)—are among the most common chronic conditions in childhood and adolescence. Together, they constitute a major group of noncommunicable diseases that have increased substantially in prevalence over the past decades, particularly in industrialized and urbanized regions. Current estimates suggest that 10%–30% of the global population is affected [1, 2]. In Germany, epidemiological data demonstrate that one in six children (16.1%) is diagnosed with at least one of the three major atopic diseases—AD, AR, or asthma—amounting to more than 2.1 million affected children and adolescents [3]. The 12‐month prevalence is reported at 7.0% for AD, 3.5% for asthma, and 8.8% for AR [3]. The prevalence of FA seems to have plateaued in the last 10 years in Germany at about 3% of children. FA is often suspected by parents but only rarely diagnosed by oral food challenge [4].

These conditions are not only common but also highly burdensome. They are typically chronic, show relapsing courses, and often coexist. Their consequences extend far beyond the acute symptoms, leading to sleep disturbances, impaired school performance, social exclusion, reduced participation in daily activities, and significantly diminished health‐related quality of life for patients and families alike. From a societal perspective, atopic diseases impose considerable costs on healthcare systems through repeated physician visits, hospitalizations, pharmacotherapy, and indirect costs related to parental work absence. Longitudinal analyses further emphasize that allergic and atopic diseases frequently persist into adulthood, creating cumulative long‐term burden if not diagnosed and managed early. In several European countries and beyond, prevention programs have been or are currently initiated to reduce personal and societal burden for chronic diseases such as atopy [5, 6, 7].

### 1.2 Natural Development of the Diseases

Multifactorial interactions between host and environmental factors drive the development and course of atopic diseases. Genetic predisposition plays a crucial role, as family history of atopy markedly increases the likelihood of disease development. However, gene–environment interactions, epigenetic modifications, and microbiome‐related mechanisms are increasingly recognized as decisive modulators of risk. Dysbiosis of gut and skin microbiota, urban lifestyle, reduced exposure to biodiversity, and westernized dietary patterns all contribute to maladaptive immune responses.

Allergic conditions can develop singularly, as sequential manifestations or in a multimorbidity framework. In a subgroup of children, AD occurs first, typically in infancy. An impaired epidermal barrier allows enhanced allergen penetration and increases the risk of food sensitization and subsequent FA. Studies show that every second child with AD is sensitized to at least one food allergen, and every third child develops clinical FA [8, 9]. Sensitization, identified by specific IgE formation, often precedes clinical disease and thus represents an important biomarker of elevated risk. However, not all children with elevated specific IgE develop clinically relevant allergic symptoms, and the poor specificity of this biomarker, especially for the prediction of food allergy, prohibits general population‐wide screening for IgE in the absence of clinical indications to avoid causing uncertainty among families and unnecessary diets or other therapies. Later in childhood, AR frequently develops, and AR itself is one of the strongest predictors for asthma onset in adolescence. While causal and temporal relationships are still studied, multimorbidity adds up to a substantial burden of disease. With timely recognition and intervention, development of atopic diseases and progression can be addressed at an early time point.

### 1.3 Preventive Options

Prevention of atopic diseases can be achieved on three levels: primary, secondary, and tertiary prevention.

*Primary prevention* aims to reduce the incidence of sensitization and initial disease manifestation. The German S3 guideline on allergy prevention, developed by several scientific societies from Germany, Austria and Switzerland under the leadership of the German Society for Allergology and Clinical Immunology (DGAKI) and the Society for Pediatric Allergology (GPA), summarizes several strategies. During pregnancy, avoidance of harmful exposures such as tobacco smoke, maintenance of a balanced diet, and prudent use of antibiotics are recommended. In early infancy, breastfeeding is encouraged, though not primarily for allergy prevention but for overall health. Particularly strong evidence has emerged for the early introduction of allergenic foods. Landmark trials showed that early introduction of well‐cooked egg and peanut (in selected populations) substantially decreases the risk of developing the respective allergies and therefore found the way into the current guidelines [10, 11, 12, 13]. In Germany, early introduction of well‐cooked egg is recommended in every infant, whereas peanut introduction is recommended in infants with AD and regular peanut consumption in the family. These findings have transformed prevention paradigms, shifting from allergen avoidance toward fostering immune tolerance.

*Secondary prevention* seeks to halt disease progression once sensitization or early symptoms are present. A key intervention is allergen immunotherapy (AIT), which has a strong evidence base in children with AR. Several prospective studies have demonstrated that initiating AIT early not only alleviates symptoms and reduces medication use but also lowers the risk of progression to asthma [14]. Early treatment of AD may also modify the risk of developing FA, although robust evidence is still lacking [15, 16].

*Tertiary prevention* focuses on preventing exacerbations and comorbidities in children with established allergic disease. Effective control of asthma with anti‐inflammatory therapy, structured management of AD, and guideline‐based interventions in FA and AR are critical to avoid long‐term complications. Importantly, cost‐effectiveness studies highlight that early causal treatments such as AIT are not only clinically beneficial but also economically advantageous, as they reduce the likelihood of severe chronic disease courses and associated costs [17, 18].

### 1.4 Role of Atopic Diseases in Current Early Assessment Programs

Despite their prevalence and societal impact, atopic diseases are largely absent from existing pediatric preventive frameworks. In Germany, standardized preventive health check‐ups (the “U examinations,” documented in the yellow booklet) monitor growth, neurodevelopment, and general health, but do not contain systematic assessments of allergy risk. No structured questions about family history of atopy, environmental exposures, or early allergic symptoms are included. Consequently, children at elevated risk are rarely identified until overt disease develops, at which point preventive opportunities may already have been missed.

International experience underlines the benefits of systematic, population‐wide approaches. The Finnish Allergy Programme (2008–2018) pioneered a paradigm shift from allergen avoidance to tolerance promotion and emphasized early recognition and timely treatment of allergic conditions. The program certifying and auditing 31 allergy centers that together performed ~90% of all national testing demonstrated that coordinated public health strategies can improve disease trajectories, standardize diagnostics, decrease unnecessary dietary restrictions without an increase in anaphylaxis, and reduce healthcare costs [5, 6]. Importantly, the Finnish approach integrated allergy care into child welfare clinics, schools, and community settings, thereby strengthening primary care and improving early case detection. These results strongly suggest that structured risk assessment and early interventions are feasible and beneficial at a population level. By contrast to the Finnish situation, in larger and more populous countries such as Germany, allergy testing is carried out by thousands of pediatricians, making consistent quality control more difficult and increasing the risk of indiscriminate testing.

### 1.5 Unmet Need/Objectives of This Initiative

Despite mounting evidence for effective prevention and treatment options, there is currently no comprehensive program for allergy‐focused clinical risk assessment and early recognition tools in childhood. Pediatric preventive check‐ups do not systematically address risk factors, sensitization markers, or early clinical signs of allergy. Allergy diagnostic including testing is usually only initiated once children present with substantial symptoms that impair health‐related quality of life. This reactive approach delays timely preventive and therapeutic measures.

The unmet need is therefore twofold: (i) to develop validated, standardized tools that identify children at elevated risk during routine pediatric visits; and (ii) to integrate these tools seamlessly into existing healthcare structures. A preventive care program must be multifactorial, accounting for host‐related risk factors (such as family history, genetic predisposition, microbiome alterations), environmental exposures (tobacco smoke, indoor allergens, pollution), and early clinical manifestations (eczema, recurrent wheeze, rhinitis symptoms, food intolerance). Moreover, early risk assessment and recognition of allergies in children must be age‐specific, recognizing that different atopic diseases emerge at distinct developmental stages.

The overarching objective of this initiative is to provide a structured algorithm—implemented alongside established pediatric preventive check‐ups—that reliably detects children with an increased risk of developing allergic disease. Once identified, these children can undergo targeted diagnostic evaluations, receive evidence‐based counseling, and, where indicated, be referred for further diagnostic, such as oral food challenge, or causal interventions such as AIT. By shifting from a reactive to a proactive approach, such a preventive strategy aims to improve long‐term health outcomes, reduce disease burden for families, and lower healthcare costs. The initiative thus represents an important step toward comprehensive, integrated prevention and early management of allergic diseases in childhood.

## 2 Methodological Approach

### 2.1 Systematic Literature Review

This review builds upon a systematic assessment of the current evidence base on allergy preventive care programs in childhood. A comprehensive literature search was performed in PubMed, Cochrane, and GIN databases, combining MeSH terms and free‐text keywords for allergic diseases (e.g., atopic dermatitis, allergic rhinitis, asthma, food allergy) with terms related to screening, early diagnosis, and prevention (Appendix S1). To ensure a balanced evidence synthesis, both meta‐analyses and systematic reviews were prioritized, supplemented by original research articles where appropriate. Only publications in English or German were included, and emphasis was placed on studies published within the past two decades, with preference for recent evidence from the last 5 years.

Following the initial database screening, abstracts were evaluated for eligibility by two independent reviewers, focusing on relevance to early identification of allergic diseases, risk stratification, and early detection approaches in pediatric populations. Full‐text articles were then retrieved and assessed for methodological quality with particular attention to study design, sample size, and outcome measures. Where available, data on sensitivity, specificity, feasibility, and cost‐effectiveness of strategies for screening, risk assessment and early recognition were extracted. In addition, national and international clinical practice guidelines were reviewed to identify current recommendations and gaps in routine pediatric care.

### 2.2 Task Force

In parallel with the literature review, a multidisciplinary Task Force was convened to guide the development of the proposed risk assessment and early recognition framework. The group included pediatricians, allergists, pulmonologists, dermatologists, nutrition experts, epidemiologists, and representatives of patient organizations. This composition ensured that both scientific evidence and patient‐centered perspectives were incorporated. The Task Force reviewed the evidence summaries, discussed feasibility within routine preventive care, and iteratively refined the algorithms for allergy‐focused clinical risk assessment and early recognition.

Consensus was achieved through structured discussions and alignment with existing national and international guidelines. Experiences from large‐scale public health initiatives, particularly the Finnish Allergy Programme, were integrated with emerging pilot projects in Germany (Sublingual Immunotherapy Research for Environmental & Food Allergies; https://allergyeasy.com/research‐studies/). This combined approach—systematic evidence synthesis and expert consensus—provides a robust methodological foundation for the preventive concept.

## 3 Results

### 3.1 Results of a Systematic Literature Review

The systematic review identified a substantial body of evidence addressing risk assessment and early detection of atopic diseases in children (see Appendix S1). Most publications focused on asthma and atopic dermatitis, with fewer studies specifically evaluating food allergy or allergic rhinitis. Several validated questionnaires and parental reports were found to have moderate sensitivity in identifying children at risk, particularly when combined with clinical examination. Objective measures such as skin prick testing and IgE determination were frequently discussed but are less suitable for large‐scale population screening due to costs and logistics. Screening for IgE sensitization also carries the high risk of instilling fears in families and children (yet) without IgE‐mediated symptoms/disease (“silent sensitization”) and thus recommendations of unnecessary therapies, such as diets. School‐ and community‐based programs demonstrated that structured risk assessment for allergies is feasible and can improve early diagnosis, reduce disease burden, and facilitate targeted prevention. Overall, the evidence supports that systematic identification of high‐risk children is possible and provides benefit compared to routine pediatric care.

### 3.2 Task Force Defined Algorithms for Early Risk Assessment and Recognition of Allergies in Children

#### 3.2.1 Atopic Dermatitis

Atopic dermatitis (AD) is often the first clinical manifestation, typically emerging in infancy. Several risk factors for its development have been clearly identified [19, 20, 21]. Genetic predisposition is paramount: loss‐of‐function mutations in the Filaggrin gene (FLG) markedly increase susceptibility. However, there is no robust evidence that genetic screening is helpful for the prevention of atopic disease manifestations. In two month old infants, visibly hyperlinear palms—especially when combined with elevated skin TARC/CCL17 levels—identify a subgroup at substantially increased risk of developing AD in early childhood, largely independent of filaggrin mutation status [22]. Family history of atopy—particularly parental eczema or asthma—is a strong predictor. Environmental and lifestyle factors, such as exposure to tobacco smoke, pollution, and low microbial diversity, further enhance risk. In addition, skin barrier dysfunction and early allergic sensitization are important biological markers associated with disease onset and persistence.

Within a longitudinal assessment strategy, these risk factors can be systematically identified during routine pediatric preventive visits. A structured parental questionnaire at each regular visit captures family history, perinatal exposures, and early environmental influences. Clinical inspection by the pediatrician allows recognition of first signs of dry skin, eczematous lesions, or recurrent itching. When symptoms appear, standardized scoring tools such as the SCORAD or the Patient‐Oriented Eczema Measure (POEM) can be applied to classify severity. In high‐risk infants, preventive counseling can be initiated promptly—for example, it may be helpful to promote skin barrier care with emollients and avoiding irritants.

The Task Force recommends that every well‐child visit include a brief risk assessment and targeted inspection for early AD. This simple, repeated approach ensures timely identification of children at elevated risk and facilitates early referral or intervention according to guideline‐based management. Routine genetic testing is not indicated and should not be part of pediatric preventive care. Table 1 summarizes established risk factors and predictive markers for AD.

**TABLE 1:** Proposed items for risk assessment and early identification of atopic dermatitis in children.

Domain	Assessment/question	Timing (U2–U9 visits^a^)
Family history	Parental/sibling history of AD, asthma, or allergic rhinitis	U2, updated at each visit
Genetic/biological risk	Children with known hereditary skin barrier defects (e.g., FLG mutation), if already documented, may have increased risk	At birth/first visit—routine genetic testing is not indicated
Environmental exposure	Tobacco smoke at home, urban vs. rural living, pollutant exposure	U2, updated regularly
Early skin symptoms	Dry skin, recurrent itching, first eczematous lesions, visibly hyperlinear palms	Every visit (clinical exam)
Sensitization markers	Early food reactions, signs of clinically relevant allergic sensitization (parental report, allergy tests)	U4–U9 only to substantiate a specific justified suspicion of a clinically relevant allergy, no testing of asymptomatic childre
Preventive counseling	Use of emollients for dry skin, avoidance of irritants, early parental guidance	Initiated if risk factors present

^a^ U2: 3–10 days of life; U3: 4–5 life week; U4: 3–4 months; U5: 6–7 months; U6: 10–12 months; U7: 21–24 months; U8: 46–48 months; U9: 60–64 months.

#### 3.2.2 Food Allergy

Food allergy (FA) is among the earliest manifestations of atopic diseases, often presenting during infancy with reactions, for example, to cow's milk, hen's egg, peanut, or tree nuts. Its prevalence in high‐income countries has increased significantly over the past decades [23], and FA now represents one of the most important health concerns in early childhood. The disease is associated not only with acute risks such as anaphylaxis but also with substantial psychosocial burden for children and their families.

##### 3.2.2.1 Risk Factors and Predictive Markers

Several risk factors for FA development have been identified. Genetic predisposition plays an important role: children with a parental history of atopy are at increased risk. Atopic dermatitis (AD), particularly when early and severe, is one of the strongest predictors of FA, reflecting the impaired skin barrier and enhanced transcutaneous sensitization [24]. Early allergic sensitization, documented by skin prick testing or specific IgE, is a marker for increased risk for subsequent FA, especially in polysensitized infants [8]. Whereas the sensitivity of specific IgE testing to food allergens is high, the specificity is poor, and the risk for causing uncertainties in the families and recommending unnecessary diets thus is high. Pediatricians should be supported in reassuring parents and actively discouraging unnecessary testing [25, 26]. Environmental exposures such as tobacco smoke or urban pollution may further modulate the risk, while breastfeeding and early nutritional exposures influence disease trajectory in complex ways [27, 28, 29, 30].

Predictive markers found in the literature include eczema severity, early sensitization to food or aeroallergens, and immunological signatures such as elevated IgE or eosinophils [31, 32, 33]. Birth cohort studies show that infants with early AD plus food sensitization carry a higher risk for asthma and allergic rhinitis [34].

##### 3.2.2.2 Risk Assessment and Early Recognition

Systematic FA screening has not been implemented at the population level. The Task Force developed a pragmatic algorithm for risk assessment and early recognition of FA embedded in routine pediatric preventive visits.

- *Step 1: Risk asessment and preventive counseling*: At U2 and subsequent visits, parents are asked about family history of atopy, early eczema, and previous adverse food reactions. Recommendation to introduce a wide range of foods early and to maintain regular consumption are given to all families without clinical signs of food allergy in children.
- *Step 2: Clinical evaluation*: Physicians inquire about immediate‐type symptoms (urticaria, angioedema, vomiting, and wheeze) after food ingestion.
- *Step 3: Diagnostic referral*: Only children with clear indications of allergic reactions to food should be referred for confirmatory testing (skin prick test, specific IgE, oral food challenge) according to the guidelines for diagnosis of FA. If in a child, a certain food is avoided solely because of a specific positive IgE test result, an oral food challenge should be performed as soon as possible to confirm or exclude allergy.
- *Step 4: Dietary and anaphylaxis counseling*: In children with confirmed food allergy, elimination diets and anaphylaxis training are conducted as recommended in the guidelines.

This approach of Step 1 is supported by strong evidence from interventional trials. The LEAP study demonstrated that early peanut introduction markedly reduced peanut allergy incidence by school age [10]. The EAT study confirmed the feasibility of early introduction of multiple allergenic foods but also its difficulties, while the PETIT trial showed that early heated egg introduction reduced egg allergy risk in high‐risk infants with eczema [35, 36]. More recently, the PreventADALL trial combined early allergen introduction with skin barrier protection. While the study was not able to provide evidence that regular emollient baths and facial cream from early infancy reduced food allergy, it did provide clear evidence supporting the effectiveness of early oral food introduction in reducing the risk of food allergy [37].

##### 3.2.2.3 Clinical Relevance

Early FA identification and prevention have broad implications. Unnecessary elimination diets can be avoided, nutritional deficiencies prevented, and quality of life improved. Importantly, causal interventions such as oral immunotherapy (OIT) are now available in specialized centers and may alter disease trajectory. Integrating FA risk assessment and early recognition into pediatric routine visits provides a feasible and cost‐effective opportunity to identify high‐risk infants, deliver preventive counseling, and initiate appropriate referral. Table 2 summarizes established risk factors and predictive markers for FA.

**TABLE 2:** Proposed items for early identification of food allergy in childhood.

Domain	Assessment/question	Timing (U2–U9 visits)
Family history	Parental/sibling history of FA, AD, asthma, or AR	U2, updated each visit
Atopic dermatitis	Presence and severity of AD (mild/moderate/severe)	Every visit (clinical exam)
Early food reactions	History of: urticaria, angiedema, vomiting, diarrhea, wheeze associated to food ingestion	Every visit, parental report
Sensitization markers	Justified suspicion of clinically relevant early allergic sensitization (parental report, prior test results)	U4–U9 only to substantiate a specific justified suspicion of a clinically relevant allergy, no testing of asymptomatic children
Environmental exposure	Tobacco smoke at home, delayed introduction of allergenic foods, low microbial contact	U2, updated regularly
Preventive counseling	Advice on early dietary introduction, avoidance of unnecessary restrictions, emollient use	Initiated if risk present
Referral criteria	Severe reactions, multiple risk factors, or persistent symptoms → referral to specialist	As indicated

#### 3.2.3 Asthma

Asthma is one of the most common chronic diseases in children. It typically develops in preschool or early school years and is characterized by recurrent wheezing, cough, and variable airflow limitation. The disease imposes a major burden on quality of life, school performance, and healthcare utilization.

##### 3.2.3.1 Risk Factors and Predictive Markers

Asthma development is shaped by risk factors and predictive markers. Risk factors are exposures or traits increasing disease likelihood, whereas predictive markers identify children most likely to develop persistent asthma.

Risk factors include family history of asthma or atopy, male sex (for early childhood asthma), preterm birth, passive smoking, air pollution, and viral infections [38, 39, 40]. Severe bronchiolitis due to rhinovirus or respiratory syncytial virus (RSV) in infancy substantially increases later asthma risk [41]. Recurrent wheezing in early life is another important risk indicator [42].

Predictive markers provide higher prognostic accuracy. Early allergic sensitization, especially polysensitization, is the strongest predictor [43, 44]. Elevated blood eosinophils and high FeNO levels indicate type 2 inflammation and correlate with persistent phenotypes [45]. Clinical tools such as the Asthma Predictive Index (API) combine symptoms (recurrent wheezing, eczema) and biomarkers (IgE against inhalant or food allergens, blood eosinophils) to estimate risk [46]. The Pediatric Asthma Risk Score (PARS) provides a more nuanced risk assessment than the API by using a weighted scoring system and accounting for additional factors such as ethnicity and polysensitization [47]. Wheeze pattern is of limited predictive value for the development of asthma over time. The ERS Taskforce explicitly notes that episodic viral and multitrigger wheeze “are not very informative in predicting response to treatment and are not stable” and therefore questions their clinical usefulness for prognostic or therapeutic decision‐making [48]. Similarly, recent reviews stress that “phenotype determined by symptom pattern alone will not remain stable” and emphasize that management should instead be guided by biomarkers such as aeroallergen sensitization or blood eosinophils [49, 50]. The strongest current predictor for progression is a combination of multiple aeroallergen sensitization, acute attacks of wheeze (exacerbation and hospitalization), and exposure to tobacco smoke before 3 years of age [51].

##### 3.2.3.2 Risk Assessment and Early Recognition

A longitudinal assessment strategy can be integrated into preventive visits:

- *Risk assessment*: Family history, perinatal factors, and environmental exposures.
- *Symptom evaluation*: Wheezing, cough, shortness of breath, nocturnal symptoms, and exercise limitation.
- *Predictive testing*: In high‐risk children, sensitization testing and eosinophil counts help risk stratification. Food mix sensitization testing should not be performed solely to satisfy the scoring requirements. In symptomatic children, sensitization testing and eosinophil counts should not be regarded as screening, but as necessary for appropriate disease characterization and management.
- *Referral*: Referral is not routinely required at earlier stages (GINA steps 1–3) provided that spirometry is available and performed correctly. Based on clinical features and basic laboratory results, referral to a child pulmonologist for further management may be indicated and is recommended for more severe asthma forms (GINA steps 4–5).

Repeated assessments during preschool years capture dynamic disease evolution. Early diagnosis enables guideline‐based therapy and preventive measures.

##### 3.2.3.3 Clinical Relevance

Early asthma identification is crucial. Prompt initiation of inhaled corticosteroids and targeted counseling improves control and may reduce exacerbations. Allergen immunotherapy in sensitized children prevents progression and decreases burden. Structured risk assessment and early recognition help distinguish transient wheezers from those at risk of persistent asthma, enabling efficient healthcare allocation. Table 3 summarizes established risk factors and predictive markers, while Table 4 outlines proposed assessment and questions for integration into well‐child visits.

**TABLE 3:** Established risk factors and predictive markers for childhood asthma.

Domain	Risk factor/predictive marker	Type	Evidence strength
Family history/genetics	Parental asthma, maternal atopy, 17q21 locus SNP	Risk factor	High (routine genetic testing is not indicated)
Early wheeze pattern	Recurrent viral‐induced wheeze, persistent wheeze phenotype	Predictive marker	Low‐to‐moderate by itself and not stable over time
Atopic sensitization	Early sensitization to aeroallergens, polysensitization	Predictive marker	High; no testing of asymptomatic children
Early wheeze pattern and multiple aeroallergen sensitization	Persistent wheeze phenotype with exacerbation together with multiple aeroallergen sensitization and tobacco smoke exposure	Predictive marker	Moderate strength together with multiple aeroallergen sensitization, acute exacerbation of wheeze and exposure to tobacco
Atopic comorbidities	Presence of AD or AR in early life	Risk factor	Moderate
Environmental exposures	Passive smoking, air pollution, indoor allergens, RSV/rhinovirus infection	Risk factor	High
Biomarkers	Elevated eosinophils, sensitization, high FeNO, positive API/PARS	Predictive marker	Moderate–high

**TABLE 4:** Proposed items for early identification of asthma in childhood.

Domain	Assessment/question	Timing (U2–U9 visits)
Family history	Parental asthma, maternal atopy	U2, update at each visit
Early wheeze	≥ 3 episodes of wheeze, especially viral‐triggered	Every visit, parental report
Respiratory symptoms	Recurrent cough, nocturnal cough, shortness of breath, exertional dyspnea	Every visit, parental report
Atopic comorbidities	Presence of AD, AR, or FA	Every visit
Environmental exposures	Tobacco smoke, indoor allergens, pollution, daycare attendance	U2, update regularly
Predictive scores	Application of API or PARS in children with recurrent wheeze	U6–U9 as indicated
Clinical evaluation	Auscultation, growth assessment, activity tolerance, spirometry (≥ 5 years) as second step	Every visit (where feasible)
Referral criteria	Persistent/recurrent wheeze, high‐risk profile, poor symptom control → referral	As indicated

##### 3.2.3.4 Experimental Options

Emerging approaches may further enhance early asthma prediction. These include in‐depth symptom trajectories in infancy as well as multi‐omics profiling (genomics, metabolomics, microbiome analyses), advanced imaging of airway inflammation, and digital health solutions such as wearables or app‐based monitoring of wheeze and cough [52, 53]. While promising, these remain experimental and require validation before routine use in pediatric risk assessment and early recognition.

#### 3.2.4 Allergic Rhinitis

Allergic rhinitis (AR) is highly prevalent in school‐aged children and adolescents and is a major risk factor for asthma development. Symptoms such as rhinorrhoea, nasal congestion, sneezing, and itching impair sleep, concentration, and health‐related quality of life. Despite its large daily impact, AR is often underdiagnosed and undertreated.

##### 3.2.4.1 Risk Factors and Predictive Markers

Risk factors include parental atopy, exposure to indoor and outdoor allergens, passive smoking, and urban living (Table 5). Early sensitization to aeroallergens strongly predicts AR development [54, 55, 56, 57]. Children with persistent rhinitis symptoms and polysensitization are at greatest risk for progression to asthma [58].

**TABLE 5:** Proposed risk assessment and early identification of allergic rhinitis in childhood.

Domain	Assessment/question	Timing (U2–U9 visits)
Family history	Parental AR, asthma, or AD	U2, update each visit
Atopic comorbidities	Presence of BA, AD or FA	Every visit
Early sensitization	Sensitization to aeroallergens (HDM, pets, pollens) if known	U4–U9 as indicated
Environmental exposure	Passive smoking, indoor allergens, urban pollution	U2, update regularly
Symptoms	Nasal obstruction, rhinorrhea, sneezing, ocular itching, seasonal patterns	U6–U9, parental/child report
Clinical examination	Nasal mucosa congestion, pale mucosa, mouth breathing	Every visit from preschool age
Referral criteria	Recurrent/persistent symptoms with comorbidities → referral for allergy testing	As indicated

Predictive markers comprise early allergic sensitization, recurrent rhinoconjunctivitis, and high IgE or eosinophil counts [59, 60, 61, 62]. Questionnaires such as the Young Children Allergic Rhinitis Questionnaire [63] assess nasal and ocular symptoms, their frequency and severity, seasonal patterns, and potential triggers and facilitate early detection in primary care.

##### 3.2.4.2 Risk Assessment and Early Recognition for AR Should Be Embedded Into Preventive Visits

- *Risk assessment*: Family history of atopy, exposure to environmental triggers.
- *Symptom inquiry*: Nasal obstruction, sneezing, rhinorrhoea, itchy eyes.
- *Clinical exam*: Nasal mucosa, conjunctival changes.
- *Referral*: Children with persistent symptoms or sensitization should be referred to specialists for causal treatment or molecular allergy diagnostics

Standardized tools can support early recognition and referral.

##### 3.2.4.3 Clinical Relevance

Early AR detection allows timely initiation of pharmacotherapy (antihistamines, intranasal corticosteroids) and environmental control. More importantly, AIT provides causal treatment, improving symptoms and preventing asthma onset [14, 64]. AIT is safe and effective in young children [65], and even in infants with high risk, sublingual immunotherapy was well tolerated [66, 67].

#### 3.2.5 Summary of Algorithms for Risk Assessment and Early Allergy Diagnosis in Childhood

**FIGURE 1:**
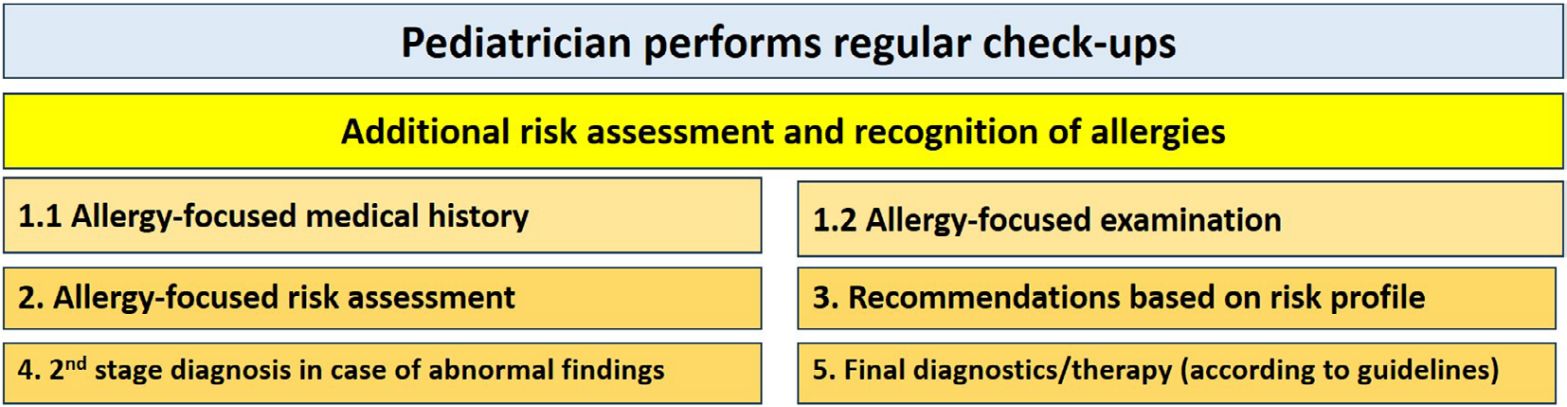
Early risk assessment and recognition of allergies in children—Basic process. The pediatrician performs regular check‐ups (Germany: U2–U9). The following additional allergy‐focused procedures are performed: (1) Specific allergological history and physical examination; (2) Assessment of atopy risk; (3) Recommendations for further allergological diagnostics and treatment; (4) If abnormal findings are detected, the pediatrician immediately initiates a second diagnostic step; and (5) Implementation/initiation of measures if appropriate findings are detected.

The Task Force defined disease‐specific algorithms for the four major pediatric atopic conditions—atopic dermatitis, food allergy, asthma, and allergic rhinitis. Each algorithm builds on a common framework: repeated risk assessment, structured symptom evaluation, targeted clinical examination, and defined referral criteria (Figure 1). This longitudinal approach ensures that children at elevated risk are identified early and consistently across the first years of life.

**FIGURE 2:**
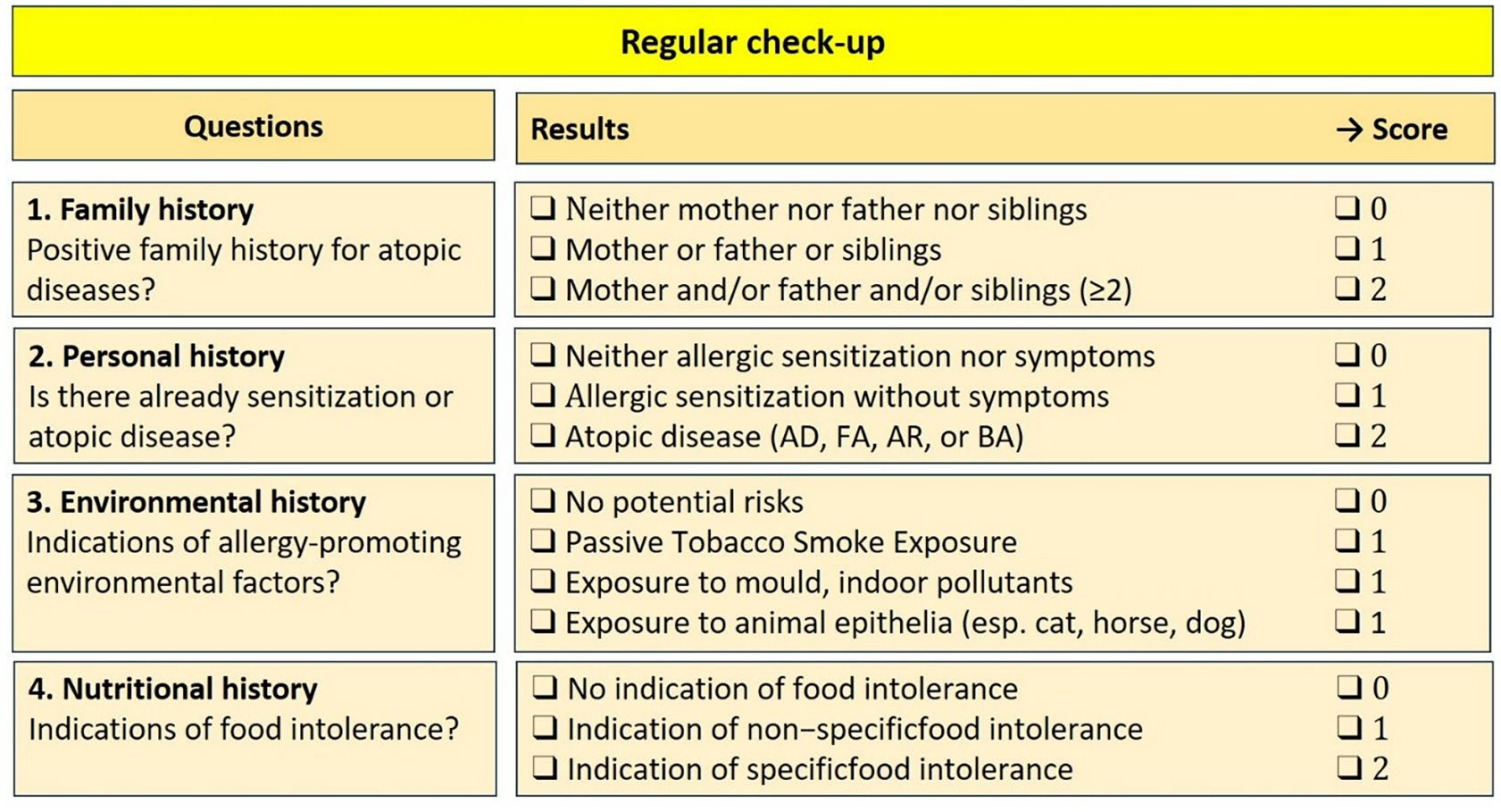
Early risk assessment of allergies in children—Medical history. At each check‐up, a basic questionnaire is filled out for the early detection of an increased risk of atopic diseases, including (1) family and (2) personal history of atopic diseases, (3) environmental history of allergen and pollutant exposure, and (4) dietary history with regard to indications of intolerances.

**FIGURE 3:**
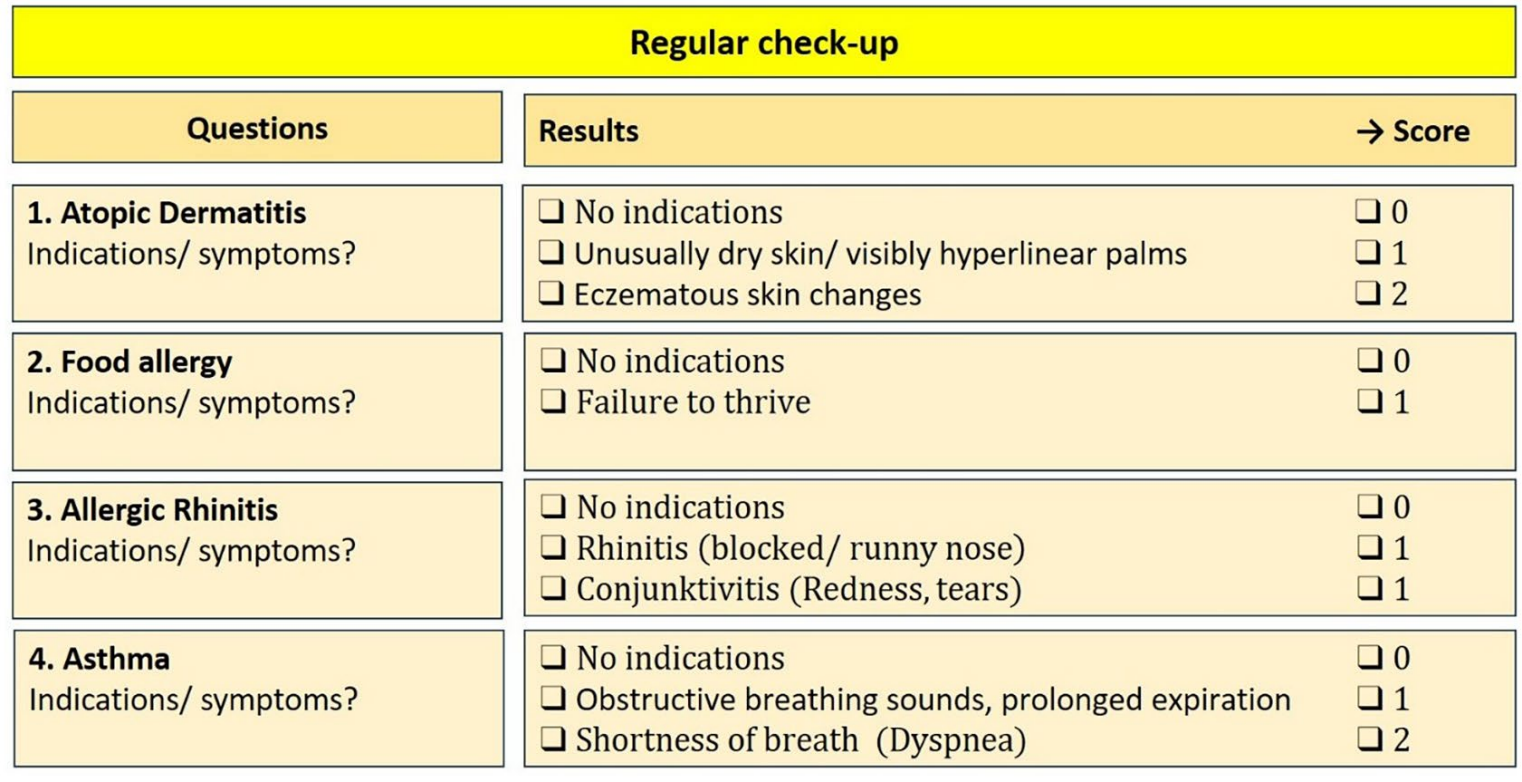
Early recognition of allergies in children—Physical examination. At each check‐up, a basic examination for symptoms of atopic diseases (atopic dermatitis (AD), food allergy (FA), allergic rhinitis (AR), or asthma (BA)) is carried out.

Across all conditions, family history of atopy remains a central element, captured at the earliest preventive visits and updated regularly (Figure 2). Environmental exposures such as passive smoking, urban air pollution, and indoor allergens were integrated as modifiable risk factors. Atopic comorbidities (AD, FA, AR, asthma) are consistently documented, recognizing their interdependence (Figure 3). In addition, validated predictive markers and scores (e.g., sensitization profiles, API/PARS for asthma) are included to guide individualized risk stratification.

**FIGURE 4:**
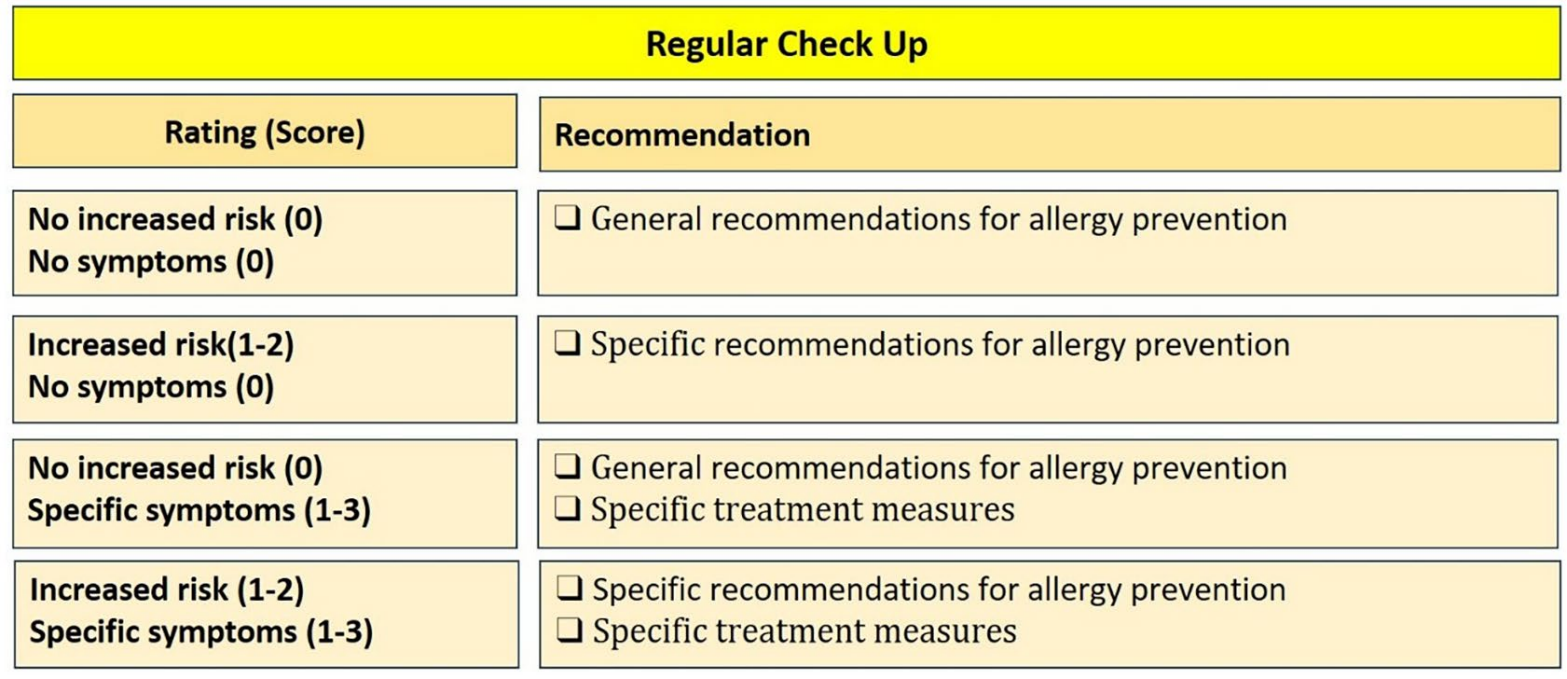
Early risk assessment and recognition of allergies in children—Recommendations. Recommendations are issued for specific occasions and provided in writing. They are based on the results of the respective risk and symptom assessment. In cases of increased risk, the specific recommendations of the Allergy Prevention Guidelines are issued. In cases of suspected or proven allergic disease, the diagnostic and therapeutic measures of the specific guidelines are issued or implemented.

The tools for risk assessment and early recognition were deliberately designed for feasibility: simple parental questionnaires, short physician assessments, and integration into existing well‐child visits (U2–U9). Documentation in preventive care booklets and, ideally, in electronic health records facilitates continuity of risk monitoring (Figure 4). Referral pathways are standardized, ensuring that children with recurrent symptoms, multiple risk factors, or high predictive scores are directed to allergy specialists for confirmatory diagnostics and targeted interventions.

By harmonizing approaches for risk assessment and early recognition across atopic diseases, the algorithms aim to close current gaps in pediatric preventive care. Early detection and timely management not only improve individual health outcomes but also contribute to reducing the long‐term burden on families, on adolescence and adulthood, and healthcare systems overall.

#### 3.2.6 Experimental Options

While the presented algorithms focus on established risk factors and predictive markers, several experimental approaches hold promise for the future of atopy screening. Multi‐omics technologies—including genomics, epigenomics, transcriptomics, proteomics, and metabolomics—may uncover novel biomarkers with higher predictive accuracy. Microbiome analyses, particularly of mucosal sites, such as the gut, nasal cells, and airway flora, are being investigated as potential tools for risk stratification. Emerging digital health applications, such as app‐based symptom diaries, wearable devices monitoring respiratory function, or artificial intelligence‐driven predictive models, can enhance real‐time screening and individual risk prediction.

Although these approaches are not yet ready for clinical implementation, ongoing research suggests that they may complement established screening algorithms in the near future. Their integration will require validation in large prospective cohorts, careful cost‐effectiveness evaluation, and consensus on ethical and data protection frameworks.

## 4 Discussion

Allergic diseases are the most frequent chronic conditions in childhood and adolescence, and their increasing prevalence poses a substantial burden on affected families and healthcare systems. Despite advances in prevention and treatment, many children are diagnosed too late to benefit from early interventions. Integrating allergy‐focused risk assessment and early recognition into routine pediatric preventive visits offers a unique opportunity to fundamentally change this trajectory.

### 4.1 Advantages of an Integrated Program for Clinical Risk Assessment and Early Recognition of Atopic Diseases

The rationale for systematic early detection of atopic diseases is compelling [7]. Atopic diseases frequently begin in the first years of life, often manifesting as atopic dermatitis or food allergy and partly progressing to asthma and allergic rhinitis [68]. Identifying children with risk factors such as family history, early eczema, or sensitization enables targeted preventive measures and early treatment.

Current evidence demonstrates that primary prevention strategies can significantly reduce disease risk. Nutritional interventions, such as early introduction of allergenic foods, lower the incidence of peanut and egg allergy [10, 33, 34]. Skin barrier support in high‐risk infants reduces atopic dermatitis onset in some, but not all clinical trials [69], and breastfeeding may contribute to lower rates of eczema and asthma [25, 26]. Secondary or tertiary prevention strategies are critical, and avoidance of harmful exposures such as tobacco smoke and indoor pollutants remains essential.

In addition to prevention, early recognition facilitates causal treatment. Allergen immunotherapy (AIT) not only improves allergic rhinitis symptoms but can also prevent asthma onset [14]. Its efficacy and safety in young children make it an attractive intervention when initiated promptly [65, 66]. Allergy‐focused clinical risk assessment and early recognition are therefore the prerequisite to identify suitable candidates early in the disease course.

On a population level, the Finnish Allergy Programme illustrates the benefits of coordinated early management: prevalence and severity of allergic diseases decreased, quality of life improved, and healthcare costs were reduced [5, 70]. Integrating allergy‐focused clinical risk assessment and early recognition into existing pediatric preventive visits would allow systematic identification of at‐risk children with minimal additional effort. Standardized questionnaires and simple clinical assessments are feasible, acceptable, and can be applied across different healthcare settings.

### 4.2 Limitations (Content, Feasibility, Costs)

Potential limitations must be acknowledged. Predictive markers are not perfect, and overdiagnosis may occur. There is already an excess of diagnostic testing in the area of food allergies, and the emphasis should be on reducing unnecessary testing. It is important to note that in medical practice the term sensitization is often misinterpreted as allergy, although sensitization alone does NOT equate to clinical disease. IgE‐testing of asymptomatic children therefore should be avoided. Implementation of the assessment and detection tools requires training of healthcare professionals and careful communication to avoid parental anxiety. Feasibility depends on healthcare system structures, resources, and reimbursement models. Nevertheless, embedding allergy‐focused clinical risk assessment and early recognition into established preventive care minimizes additional burden, and costs are likely offset by long‐term savings through reduced morbidity.

### 4.3 Economic Impact Modeling

Economic models consistently indicate that early detection and prevention of allergic diseases can lead to substantial savings by reducing medication use, hospitalizations, and productivity loss [71, 72]. Pilot studies in different healthcare systems are now required to validate cost‐effectiveness and guide broad implementation.

## 5 Summary and Conclusions

Atopic diseases are the most common chronic conditions in childhood and continue to increase worldwide. They often begin early in life and can progress from atopic dermatitis and food allergy to asthma and allergic rhinitis, or present in parallel. Despite their high prevalence and burden, many children are still diagnosed late, missing opportunities for timely prevention and causal interventions.

This review highlights the rationale and feasibility of introducing a structured program for clinical risk assessment and early recognition of atopic diseases in childhood. Evidence from epidemiological studies, intervention trials, and national programs such as the Finnish Allergy Programme underscores that early identification of at‐risk children allows targeted preventive counseling, timely allergen introduction, and consideration of allergen immunotherapy where appropriate. Such measures can alter the natural history of allergic disease, improve health‐related quality of life, and reduce long‐term health care costs.

In conclusion, integrating allergy‐focused clinical risk assessment and early recognition into routine pediatric preventive care offers a promising strategy to reduce the burden of atopic diseases. Future research and pilot projects are required to refine the algorithms, assess cost‐effectiveness, and ensure equitable access for all children (Boxes 1 and 2).

BOX 1 **Future research perspectives.**

**Table:** 

Open questions and priority areas for advancing early risk assessment and recognition of allergies in children. Validation of assessment tools in prospective population‐based cohorts: Assess feasibility, predictive value, and long‐term outcomes in real‐world settings. Integration of multi‐omics approaches: Explore genomic, epigenomic, transcriptomic, and microbiome markers to improve individual risk prediction. Development of digital health tools: Evaluate app‐based symptom diaries, wearable sensors, and AI‐supported decision aids for early detection and monitoring. Assessment of cost‐effectiveness and health economic impact: Model short‐ and long‐term benefits of structured allergy‐focused clinical risk assessment and early recognition in different healthcare systems. Ethical and psychosocial implications of early risk assessment and recognition: Ensure informed consent, minimize overdiagnosis, and address potential parental anxiety. Optimization of skin barrier interventions: Identify infants most likely to benefit from early emollient use and refine application strategies. Expansion of causal treatments in early life: Evaluate safety, efficacy, and timing of allergen immunotherapy and oral immunotherapy in infants and toddlers.

BOX 2 **Major milestone discoveries.**

**Table:** 

Key developments that have shaped current understanding and management of atopic diseases in childhood. Early allergen introduction (LEAP, PETIT, EAT studies): Paradigm shift from allergen avoidance to immune tolerance induction in infancy, now reflected in international prevention guidelines. Recognition of the “atopic march”: Understanding that early manifestations such as atopic dermatitis and food sensitization may precede asthma and allergic rhinitis. Development of the Asthma Predictive Index (API) and Pediatric Asthma Risk Score (PARS): Clinical tools for early asthma risk stratification in preschool children. Introduction of allergen immunotherapy (AIT) as **a** disease‐modifying intervention: Evidence for prevention of asthma development and long‐term symptom control in allergic rhinitis. Implementation of national prevention programs (e.g. Finnish Allergy Programme): Demonstrated feasibility and benefit of integrated early intervention strategies at a population level. Identification of genetic risk variants (e.g. FLG mutations): Established link between impaired skin barrier function and increased risk for allergic sensitization and food allergy. Emergence of type 2 inflammation biomarkers (e.g. FeNO, eosinophils): Enabled phenotypic disease classification and personalized treatment approaches in pediatric asthma

## Author Contributions

All authors contributed to the conception and design of this work. E. Hamelmann performed the systematic literature search. E. Hamelmann, Bianca Schaub, and Christian Vogelberg drafted the initial algorithms for allergy‐focused clinical risk assessment and early recognition. All authors participated in manuscript drafting and critical revision. E. Hamelmann had primary responsibility for final content. All authors approved the final version of the manuscript.

## Funding

This work was supported by an unrestricted financial grant from the Initiative Allergiescreening Deutschland to the joined Task Force Allergy Screening of the German Society for Pediatric Allergology and Environmental Medicine (GPA) and the German Society for Allergology and Clinical Immunology (DGAKI).

## Conflicts of Interest

E. Hamelmann: ALK, AstraZeneca, DBV Technologies, GSK, Regeneron Pharmaceuticals Inc., Sanofi—speaker/consultant fees; BMBF, BMG, MAGS, G‐BA—research support; Wolff—data and safety monitoring board. B. Schaub: GSK, Novartis, Astra Zeneca, Regeneron Pharmaceuticals Inc., Sanofi—speaker/consultant fees; BMBF: German Center for Child and Adolescent Health (DZKJ; LMU/LMU Klinikum: 01GL2406A), German Center for Lung Research (DZL), DZL 82DZL033C2, Combat Lung diseases FP4; DFG—research support; Sanofi—Data Safety Monitoring Board. S. Lau: received honoraria for lectures and advisory boards from ALK, Allergopharma, Almirall, AstraZeneca, DBV Technologies, Engelhard, Lilly, Leo Pharma, Sanofi‐Aventis, Viatris. Furthermore, funding from German Ministry of Health (BMG PedNet Long Covid), German Research Foundation (DFG Food@ 339) and Einstein Stiftung (Berlin University Alliance CliWaC). K. Beyer: Aimmune, Danone, DBV, Hipp, Hycor, Infectopharm, Nestle, Novartis—research support; Aimmune, Akademie Fresenius, ALK, Danone, HAL, Infectopharm, Kantar Health, Primus Consulting, Nestle, Novartis, Stallergenes, ThermoFisher, Viatris—speaker/consultant fees; ALK—data and safety monitoring board. K. Blümchen: received honoraria for lectures and advisory boards from Aimmune Therapeutics, DBV Technologies, Novartis, Allergy therapeutics, Bencard Allergie, Stallergenes Greer, Thermofisher Scientifc, Danone, Allergopharma, Mylan, Sanofi, Engelhard, ALK, HAL, Celltrion, Siemens healtheneer. Furthermore, funding for research projects from Aimmune Therapeutics, DBV Technologies, Novartis, Allergy therapeutics, Nestle, Federal Ministry of Education and Research (CALM QE). M. V. Kopp: Infectopharm, Allergopharma speaker/consultant fees; BMBF: German Center for Lung Research (DZL). T. Spindler: ALK, Orion Pharma speaker fee; German Pharmacy Board: speaker, consultant. C. Vogelberg: ALK, Allergopharma, AstraZeneca, DBV Technologies, LETI, Novartis, Orion Pharma, Sanofi Aventis, Thermo Fisher speaker/consultant fees; Boehringer Ingelheim research support; Allergy Therapeutics data safety monitoring board. S. M. Schmidt: ALK, Infectopharm, Novartis, Sanofi, Vertex consultant/speaker fees.

## Supporting information


**Appendix S1:** all70224‐sup‐0001‐AppendixS1.docx.

## Data Availability

The data that support the findings of this study are available in the Supporting Information of this article.
