# Supplementary material for: Early Risk Assessment and Recognition of Allergies in Children: Rationale, Methodology, and Proposed Algorithms
Source: Allergy. 2026 Jan 25;81(6):1971–84. doi: 10.1111/all.70224 (PMC13256288; doi:10.1111/all.70224)
Supplement: Supplementary file 1 — Appendix S1: all70224‐sup‐0001‐AppendixS1.docx. [file ALL-81-1971-s001.docx]

1. PubMed, Cochrane, and GIN databases were searched using the following keywords:

((((("Dermatitis, Allergic Contact"[Mesh] OR "Nut and Peanut Hypersensitivity"[Mesh]) OR ( "Rhinitis, Allergic, Seasonal"[Mesh] OR "Rhinitis, Allergic"[Mesh] OR "Rhinitis, Allergic, Perennial"[Mesh] OR "Conjunctivitis, Allergic"[Mesh] OR "Hypersensitivity"[Mesh] OR "Nut Hypersensitivity"[Mesh] OR "Peanut Hypersensitivity"[Mesh] OR "Wheat Hypersensitivity"[Mesh] OR "Egg Hypersensitivity"[Mesh] OR "Milk Hypersensitivity"[Mesh] OR "Respiratory Hypersensitivity"[Mesh] OR "Dermatitis, Atopic"[Mesh] )) OR (((((((((((((((((((allergic sensitization[Title/Abstract]) OR (allergic sensitisation[Title/Abstract])) OR (allergic symptom[Title/Abstract])) OR (allergic rhinitis[Title/Abstract])) OR (allergic rhinoconjunctivitis[Title/Abstract])) OR (hayfever[Title/Abstract])) OR (hay fever[Title/Abstract])) OR (atopic eczema[Title/Abstract])) OR (atopic dermatitis[Title/Abstract])) OR (allergic asthma[Title/Abstract])) OR (food allergy[Title/Abstract])) OR (cow´s milk allergy[Title/Abstract])) OR (peanut allergy[Title/Abstract])) OR (soy allergy[Title/Abstract])) OR (soy hypersensitivity[Title/Abstract])) OR (wheat allergy[Title/Abstract])) OR (egg allergy[Title/Abstract])) OR (tree nut allergy[Title/Abstract])) OR (fish allergy[Title/Abstract]))) OR (((((((nut hypersensitivity[Title/Abstract]) OR (peanut hypersensitivity[Title/Abstract])) OR (cow´s milk hypersensitivity[Title/Abstract])) OR (wheat hypersensitivity[Title/Abstract])) OR (egg hypersensitivity[Title/Abstract])) OR (milk hypersensitivity[Title/Abstract])) OR (respiratory hypersensitivity[Title/Abstract]))) AND ("Mass Screening"[Mesh] OR "Neonatal Screening"[Mesh] OR "Early Diagnosis"[Mesh] OR early detection[Title/Abstract] OR questionnaire[Title/Abstract] OR early diagnosis[Title/Abstract] OR screening[Title/Abstract] OR neonatal screening[Title/Abstract] OR mass screening[Title/Abstract])) AND (meta-analysis[Publication Type] OR meta-analysis[Title/Abstract] OR meta-analysis[MeSH Terms] OR review[Publication Type] OR search*[Title/Abstract]).


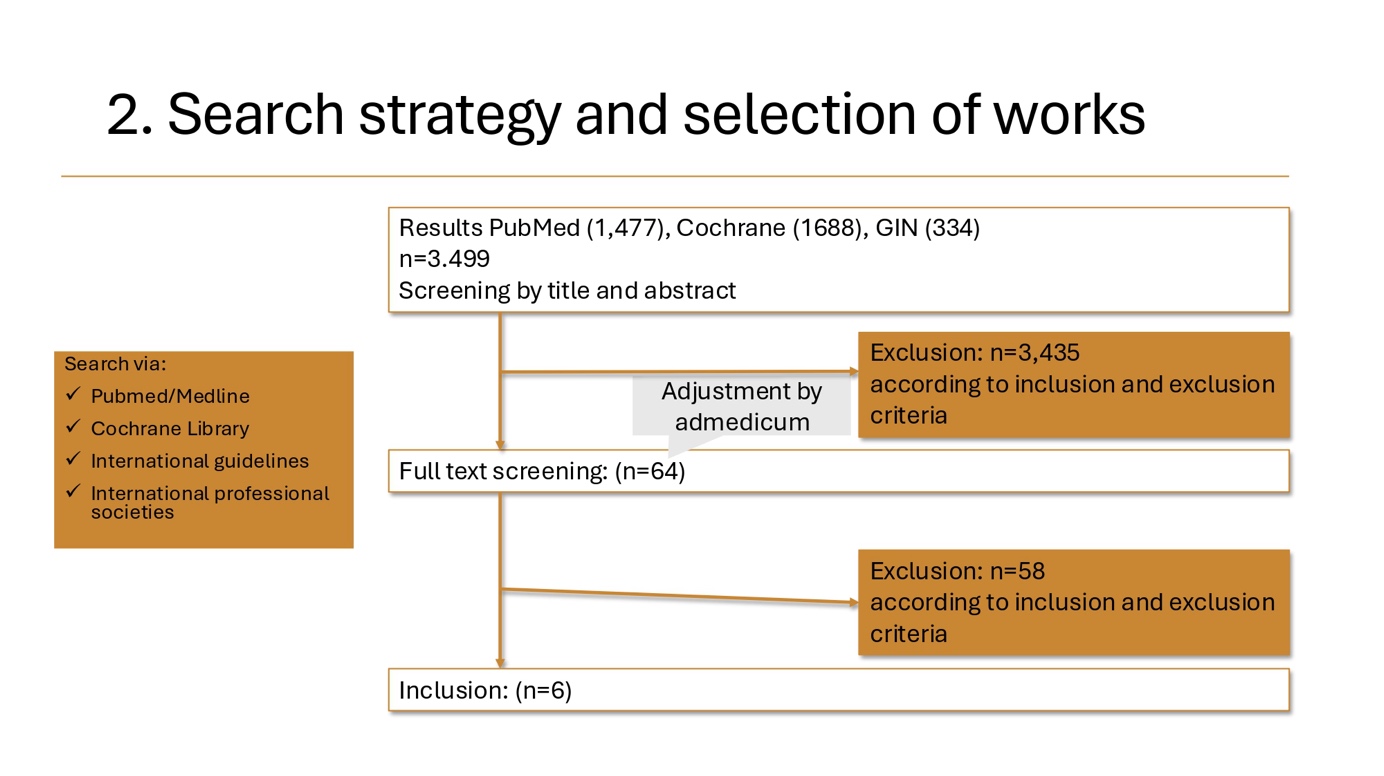


Literature included after search:

1. Bruzzese, Jean-Marie; Evans, David; Kattan, Meyer (2009): School-based asthma programs. In: The Journal of allergy and clinical immunology 124 (2), S. 195–200. DOI: 10.1016/j.jaci.2009.05.040.
2. Chamlin, Sarah L.; Kaulback, Kellee; Mancini, Anthony J. (2009): What is "high risk?" a systematic review of atopy risk and implications for primary prevention. In: *Pediatric dermatology* 26 (3), S. 247–256. DOI:10.1111/j.1525-1470.2008.00807.x.
3. Cropper, J. A.; Frank, T. L.; Frank, P. I.; Laybourn, M. L.; Hannaford, P. C. (2001): Respiratory illness and healthcare utilization in children: the primary and secondary care interface. In: *The European respiratory journal* 17 (5), S. 892–897. DOI: 10.1183/09031936.01.17508920.
4. Gerald, Lynn B.; Sockrider, Marianna M.; Grad, Roni; Bender, Bruce G.; Boss, Leslie P.; Galant, Stanley P. et al. (2007): An official ATS workshop report: issues in screening for asthma in children. In: *Proceedings of the American Thoracic Society* 4 (2), S. 133–141. DOI: 10.1513/pats.200604-103ST.
5. Klok, Ted; Ottink, Mark D.; Brand, Paul L. P. (2020): Question 6: What is the use of allergy testing in children with asthma? In: *Paediatric respiratory reviews* 37, S. 57–63. DOI: 10.1016/j.prrv.2020.07.007.
6. Remes, S. T.; Pekkanen, J.; Remes, K.; Salonen, R. O.; Korppi, M. (2002): In search of childhood asthma: questionnaire, tests of bronchial hyperresponsiveness, and clinical evaluation. In: *Thorax* 57 (2), S. 120–126. DOI: 10.1136/thorax.57.2.120.
